# Surgical treatment for thoracoabdominal intra-aortic thrombus with multiple infarctions: a case report

**DOI:** 10.1186/s13256-016-1017-1

**Published:** 2016-08-10

**Authors:** Kenichiro Uchida, Mitsuharu Hosono, Toshihiko Shibata, Daisuke Kaku, Tomonori Yamamoto, Takafumi Terada, Naoki Shinyama, Yasumitsu Mizobata

**Affiliations:** 1 Department of Traumatology and Critical Care Medicine, Osaka City University Graduate School of Medicine, 1-4-3 Asahi-machi, Abeno-ku, Osaka City, Osaka 545-8585 Japan; 2 Department of Cardiovascular Surgery, Osaka City University Graduate School of Medicine, 1-4-3 Asahi-machi, Abeno-ku, Osaka City, Osaka 545-8585 Japan

**Keywords:** Intra-aortic thrombus, Infarction, Surgical thrombectomy, Erythrocytosis

## Abstract

**Background:**

Mobile intra-aortic thrombus without atherosclerosis, aneurysm, or congenital coagulopathy is very rare, and there are few reports especially in young or middle-aged patients. Furthermore, there are presently no established guidelines or common strategies for the treatment of mobile intra-aortic thrombus.

In this case report, we describe the first case of intra-aortic thrombus caused by secondary erythrocytosis and describe the recommended treatment strategy for intra-aortic thrombus.

**Case presentation:**

We report a case of an independent 40-year-old Asian man with a current history of heavy cigarette smoking who had sudden onset of abdominal and lumbar pain. Contrast-enhanced computed tomography revealed partial renal and splenic infarction, and he was transferred to our hospital. He also had a large mural thrombus in his thoracoabdominal aorta. Blood analysis on admission showed a hemoglobin level of 19.4 g/dL and hematocrit of 54.3 %; his international normalized ratio of prothrombin time, fibrin degradation products, and activated partial thromboplastin time levels were 1.02, 2.8 μg/ml, and 26.9 seconds respectively. We could find no abnormalities in protein C and protein S activity levels. Lupus anticoagulant and anti-cardiolipin antibody were both negative. He had no past medical history of arrhythmia and we found no signs of an arrhythmic event during admission.

We promptly started anticoagulant therapy, but as the thrombus seemed at high risk of causing further critical infarction, we performed emergency aortic thrombectomy using partial extracorporeal circulation. To prevent dissemination of the thrombus during extracorporeal circulation, we first clamped his proximal and distal aorta on either side of the thrombus just before initiating extracorporeal circulation.

After the aortotomy we removed a 14-cm length of intra-aortic thrombus without residual lesion. He was discharged from our hospital 20 days after surgery. From the results of his blood analysis, we considered the only cause of this thrombus was secondary erythrocytosis, which was probably induced by his current heavy cigarette smoking.

**Conclusion:**

We are the first to report such a thrombosis caused by secondary erythrocytosis and conclude that once the diagnosis of intra-aortic thrombus with systemic embolism is clear, emergency surgical removal of such a thrombus must be considered to prevent further embolic complications.

## Background

Mobile intra-aortic thrombus is very rare but sometimes occurs mainly due to thrombi in the left side of the heart [1]. Furthermore, there are few reports of mobile intra-aortic thrombus without atherosclerosis, aneurysm, or congenital coagulopathy, particularly in young or middle-aged patients [2].

The therapeutic strategy for mobile intra-aortic thrombus is still controversial and treatment consensus has not been established [1–3].

We report a case of a middle-aged Asian man who complained of abdominal and lumbar pain. Contrast-enhanced computed tomography (CECT) revealed a mobile thrombus in his thoracoabdominal aorta with acute renal and splenic embolism. We performed emergency surgical thrombectomy and he was discharged from hospital with no complications.

We considered that the only major cause of this thrombus was secondary erythrocytosis that was probably induced by the patient’s current heavy tobacco smoking. We could not find any similar published articles describing the same cause of a thrombus.

## Case presentation

A 40-year-old Asian man was hospitalized in our emergency department because of sudden abdominal and lumbar pain that woke him while sleeping. He had a medical history of hypertension but was not being treated with medications. He also had a current cigarette smoking history of 40 cigarettes per day for 20 years, and had no other related familial history. A physical examination showed left upper quadrant abdominal tenderness and bilateral costovertebral angle tenderness. No cardiac murmur or abnormal respiratory sounds were heard on auscultation. His blood pressure was 215/118 mmHg, heart rate was regular at 73 beats per minute, respiratory rate was 12 breaths per minute, and his body temperature was 36.8 °C.

A serial hematologic workup showed an extremely high hemoglobin level (19.4 g/dl) and hematocrit (54.3 %), but his white blood cell count and platelet count were within normal limits. No abnormal values of blood urea nitrogen, creatinine level, and hemostasis system function were found in this workup. His international normalized ratio of prothrombin time (PT-INR), fibrin degradation products (FDP), and activated partial thromboplastin time (APTT) levels were 1.02, 2.8 μg/ml, 26.9 seconds respectively. Only the level of his D-dimer was slightly increased (2.7 μg/ml).

**Fig. 1:**
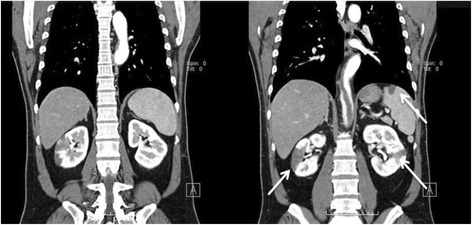
Preoperative contrast-enhanced computed tomography. The computed tomography scan showed both the multiple renal and splenic infarctions (*white arrows*) and the large intra-aortic thrombus in the descending thoracic aorta

**Fig. 2:**
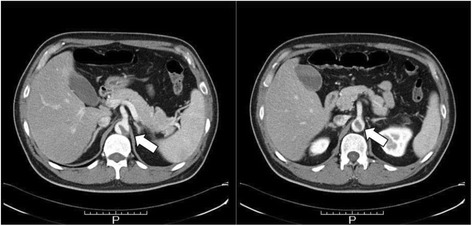
Axial view of the preoperative contrast-enhanced computed tomography. These views showed the end of the intra-aortic thrombus (*large white arrows*) indicating the likelihood of further systemic thrombosis

**Fig. 3:**
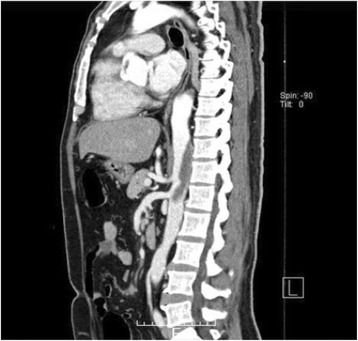
Sagittal view of the preoperative contrast-enhanced computed tomography

In a later analysis, lupus anticoagulant and anti-cardiolipin antibody were not detected, and his protein C and protein S activity levels were 115 % and 113 % respectively. His counts of erythroblasts and reticulocytes, and erythropoietin level were 0 parts per 1000, 17.2 parts per 1000, and 10.6 mIU/ml, respectively, and these results indicated no findings of polycythemia vera. His electrocardiogram was normal sinus rhythm on admission. Transthoracic echocardiography excluded wall motion abnormalities, valve dysfunction, intracardiac thrombus, and any other patent circulatory shunts. CECT revealed a huge intra-aortic thrombus at the level of the descending to abdominal aorta just superior to his renal artery with patchy emboli in his spleen and bilateral kidneys (Fig. 1) , and a segment of the thrombus existed just near the ostia of his celiac artery and superior mesenteric artery (Figs. 2 and 3).

After his admission to our emergency department, we immediately started systemic heparinization. But as we were concerned about the extremely high possibility that the thrombus could cause a further fatal systemic embolism, emergency surgical thrombectomy was performed on the same day.

The operation was started with our patient in a right half-lateral position, and preparations for femoral arteriovenous extracorporeal circulation were completed. A thoracoabdominal spiral incision was performed, and his aorta was approached at the level of the sixth intercostal via a retroperitoneal route. Epiaortic ultrasonography was performed prior to taping to detect the exact lesion of the thrombus, and we taped to aorta at the level of the fifth thoracic vertebra and just superior to the level of his renal artery. After his celiac, superior mesenteric, and left renal arteries were individually taped, his temperature was controlled to a mild hypothermia of 33 °C.

**Fig. 4:**
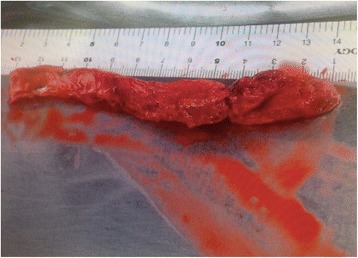
Photograph of the 14-cm length of fresh thrombus removed from the thoracic descending aorta

To prevent dissemination of the thrombus during extracorporeal circulation, we first clamped his proximal and distal aorta on either side of the thrombus, which had been preliminarily taped. After partial arteriovenous extracorporeal circulation from his femoral artery was started, an aortotomy was performed. We found a fresh thrombus and easily removed the 14-cm length of intra-aortic thrombus without residual lesion (Fig. 4). There was no arteriosclerotic lesion in the intimal layer, but a spot of organized intramural thrombus in the descending aorta was found, and fresh thrombus seemed to develop secondary to this organized intramural thrombus.

After the thrombectomy, his aorta could be sutured directly. He was discharged from our hospital 20 days after the surgery without any complications. He had no past medical history of atrial or ventricular arrhythmia and we found no signs of a paroxysmal arrhythmic event during admission.

Presently, he is still undergoing antiplatelet therapy and warfarin administration. Since he quit cigarette smoking, he has had no findings of polycythemia.

## Discussion

Most arterial embolisms occur due to thrombi in the left side of the heart [1], and only a few reports describe systemic embolism without vascular disease such as aortic dissection, aneurysm, atrial fibrillation, congenital coagulopathy, or intracardiac shunt. We only found published cases in which systemic arterial embolism occurred secondary to systemic sclerosis, malignant tumor, or protein C and protein S abnormalities [2, 3].

If intra-aortic thrombus is detected in a young patient, we have to consider and rule out congenital coagulopathy [4]. However, some reports have noted that some of these coagulopathy disorders are not detected by current multisystemic hemostatic testing methods [4, 5]. We found no abnormal hemostatic data or familial history in our patient that would cause us to suspect a hemostatic disorder, and only polycythemia secondary to his heavy cigarette smoking was considered the cause of this thrombus. Recently, tobacco smoking has been considered to be significantly related to the risk of not only venous thrombus formation but also polycythemia, platelet dysfunction, and the formation of aortic thrombus [6, 7]. Our patient also had a current history of heavy cigarette smoking and thus was at possible risk of intra-aortic thrombus formation.

For the diagnosis and assessment of intra-aortic thrombus mobility, the recommended examinations are CECT or transesophageal echocardiography (TEE) [8–10]. We performed TEE intraoperatively in our patient and confirmed the mobility of the thrombus. The probability of a thrombus that has already caused systemic embolism to cause further embolism is reported to be approximately 73 % [11], but definitive therapeutic guidelines have not been established. For now, surgical thrombectomy, endovascular therapy including the use of stents, and medical treatment with heparin or tissue plasminogen activator have been reported [1–3]. However, thrombolysis with thrombolytic agents carries the risk of selectively disseminating thrombi released into the blood stream from the stalk of the lesion. Furthermore, there is no consensus on the dose or duration of the use of thrombolytic agents or on the definitive timing of stopping thrombolysis [12–14]. We could find no reports recommending thrombolytic medication for intra-aortic thrombus that has caused systemic arterial embolism.

Recently, as remarkable endovascular devices such as stent grafts or touching balloons have been developed, some case reports have described the successful treatment of intra-aortic thrombus by endovascular therapy [15]. However, endovascular procedures for treating mobile thrombus obviously carry a high risk of iatrogenic systemic embolism [11], and as the long-term results of these devices remain to be confirmed, their appropriate adaptation for young patients such as ours is not clear [16].

If surgical thrombectomy is considered, we have to think about the risk of disseminating the remaining thrombus in the aorta at the start of extracorporeal circulation or during thrombectomy. Further, if the intimal aspect is not clear to permit direct suturing, the surgery must be extended to rebuild the aorta with a synthetic graft. However, the risk of thrombus dissemination at the start of extracorporeal circulation can be resolved by appropriate timing of the aortic clamping procedure and starting extracorporeal perfusion as described in this case.

Thrombectomy is reported to be easily completed in young patients with no arteriosclerotic vascular lesions, as in this case, and the risk of complications such as further systemic embolism or conversion to extended surgery is possibly quite less compared to that with thrombolytic medication therapy [1, 8]. Thus, surgical thrombectomy as a therapeutic strategy for intra-aortic thrombus with systemic aortic embolism has been recommended in several previous case reports [1–3, 8, 17].

The prevention of recurrence depends on the cause of the thrombus formation, and definitive evidence has not been established for either anticoagulant therapy or antiplatelet therapy [18]. However, one study reported the possibility of anticoagulant therapy offering a preventive effect [19], and our patient continues on both anticoagulant and antiplatelet therapies. To date, he has experienced no recurrent thrombosis.

## Conclusions

From the results of blood analysis of our patient, the only cause of his intra-aortic thrombus that we considered was secondary erythrocytosis that was probably induced by his current heavy cigarette smoking. We conclude that once a diagnosis of intra-aortic thrombus with systemic embolism is clear, emergency surgical removal must be considered to prevent further embolic complications.

## Abbreviations

APTT, activated partial thromboplastin time; CECT, contrast-enhanced computed tomography; FDP, fibrin degradation products; PT-INR, international normalized ratio of prothrombin time; TEE, transesophageal echocardiography
